# The Impact of Patient Age on Causes of Graft Loss After Renal Transplantation

**DOI:** 10.3389/ti.2025.14544

**Published:** 2025-05-02

**Authors:** Susanne Winkler, Min-Jeong Kim, Andrea Fisler, Stefan Farese, Felix Burkhalter, Seraina von Moos, Christian Forster, Caroline Wehmeier, Michael Dickenmann, Stefan Schaub

**Affiliations:** ^1^ Clinic for Transplantation Immunology and Nephrology, University Hospital Basel, Basel, Switzerland; ^2^ Department of Nephrology, Kantonsspital Aarau, Aarau, Switzerland; ^3^ Division of Nephology, Kantonsspital Baden, Baden, Switzerland; ^4^ Renal Division, Herz-und Nierenzentrum Aare, Solothurn, Switzerland; ^5^ Department of Nephrology, Kantonsspital Liestal, Liestal, Switzerland; ^6^ Department of Nephrology, Luzerner Kantonsspital, Lucerne, Switzerland; ^7^ Renal Division, Department of Internal Medicine, Kantonsspial Olten, Olten, Switzerland; ^8^ Molecular Immune Regulation, Department of Biomedicine, University of Basel, Basel, Switzerland; ^9^ HLA-Diagnostic and Immunogenetics, Department of Laboratory Medicine, University Hospital Basel, Basel, Switzerland

**Keywords:** renal transplantation, allograft loss, recipient age, death with graft function, allograft rejection

## Abstract

The interplay of recipient age and graft loss causes is underexplored, despite its relevance for patient management and endpoint definition in clinical trials. This study aimed to investigate the impact of recipient age on graft loss causes. In this retrospective single-center cohort study with 1743 kidney transplantations between 1995 and 2022, graft losses were assigned to either death with graft function (DwGF) or graft failure (GF). Additionally, causes of death and GF were determined by reviewing all available clinical/histological information. Data were analyzed across recipient age groups (≤40, 41–60 and >60 years) and across three time periods (1995–2004, 2005–2014, 2015–2022). Among 816 graft losses, 56% were attributed to DwGF and 44% to GF. The proportion of DwGF increased stepwise with age (21% in young vs. 52% in middle-aged vs. 76% in elderly patients; p < 0.0001), with similar proportions across the three time periods. Rejection alone or in combination with other events caused GF in 76% of young, 51% of middle-aged, and 34% of elderly patients (p < 0.0001). Main death-causes were cardiovascular events (23%), infections (23%) and malignancies (23%). Graft loss causes are strongly age-related. This might have significant implications for clinical study design and patient management.

## Graphical Abstract

**Figure:**
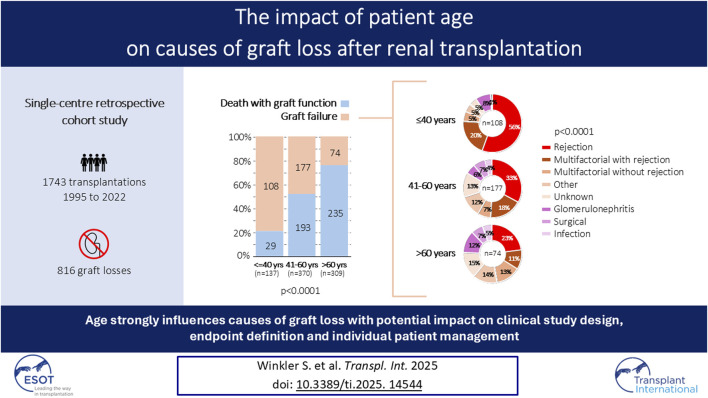

## Introduction

Over the last thirty years, significant advances in transplantation medicine have led to an improvement in both patient and death-censored renal allograft survival. Better immunological understanding and the development of more effective immunosuppressive regimens were essential for this success [1–4]. In addition, an improved management of cardiovascular diseases and their risk factors, along with overall advancements in medical care, have enabled older patients and patients with multiple comorbidities to become eligible for renal transplantation [5–7].

Nevertheless, allograft rejection and immunosuppression-related complications still remain a major challenge [8–10]. A precise understanding of the causes of graft failure and death is essential to identify areas of particular importance to further improve these key outcomes. Indeed, four studies conducted in North America and Europe in patients transplanted between 1996 and 2017 reported granular data on the causes of graft losses [10–13]. The investigators observed between 318 and 645 graft losses. Death with graft function (DwGF) accounted for 43%–59% of all graft losses, while the other 41%–57% were related to graft failure (GF). The main causes of death were cardiovascular events, infection diseases and malignancies. Among the causes of graft failure, rejection was attributed as the leading event in 12%–34%. Interestingly, Mayrdorfer et al showed in a very detailed study that rejection either as primary or secondary cause was responsible for 65% of graft failures, highlighting the ongoing significance of rejection in the current era [14].

The interplay of recipient age and the causes of graft loss is poorly explored with conflicting results, and most analyses were performed in cohorts transplanted before the year 2000 [15]. However, it might be relevant to define age-dependent endpoints in clinical trials rather than a “one-size-fits-all” approach for all patients. Therefore, the aim of this study was to investigate the impact of recipient age on the causes of 816 graft losses among 1743 transplantations performed at the University Hospital Basel from 1995 to 2022. Furthermore, we explored the evolution of patient and death-censored graft survival in different age groups over time.

## Materials and Methods

### Patient Population and Study Design

**FIGURE 1:**
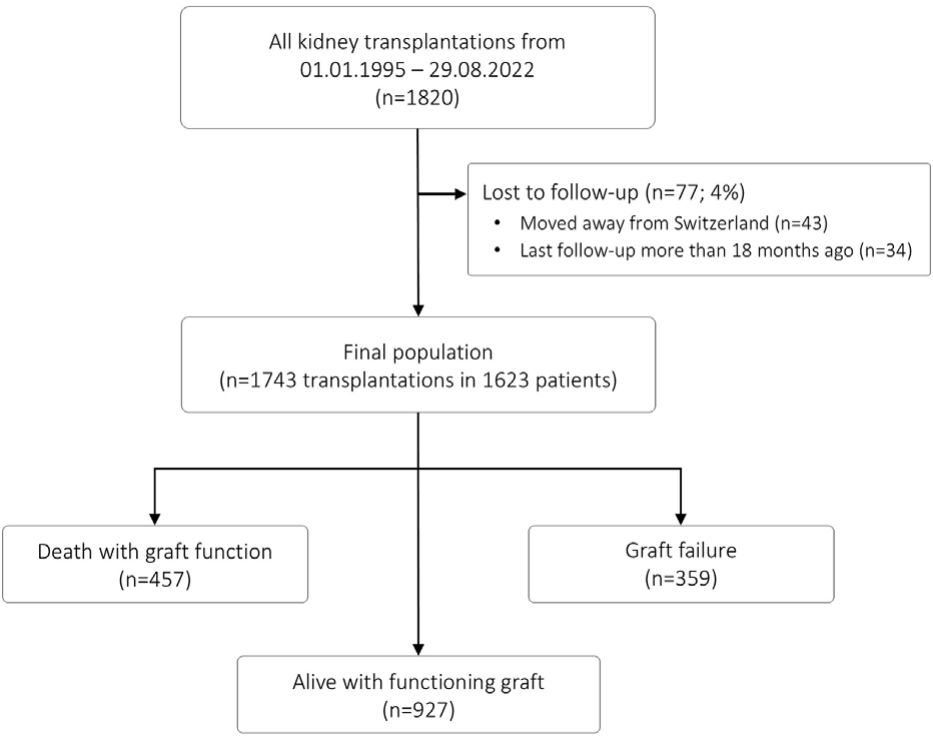
Patient flow.

This retrospective observational cohort study was approved by the local ethics committee (EKNZ 2023-01992). The patient flow is summarized in Figure 1. All adult and pediatric patients who underwent kidney transplantation at the University Hospital Basel between 1st January 1995 and 29th August 2022 were eligible. From a total of 1820 transplantations, we excluded patients without recent follow-up data (n = 77; 4%), resulting in a final population of 1743 transplantations in 1623 patients. End of follow-up was September 2023, and all transplantations had a minimal follow-up time of 1 year.

To describe the evolution of patient age as well as patient and graft survival over time, we divided the study population into three eras. These time frames correspond to major developments in transplantation medicine. The first era (1995–2004; n = 570) was dominated by Cyclosporine-based maintenance immunosuppression and immunological risk stratification based on complement-dependent cytotoxicity crossmatches. The second era (2005–2014; n = 593) was characterized by Tacrolimus-Mycophenolate (Tac-MMF) based immunosuppression and the implementation of an ABO-incompatible living donor kidney transplantation program [16]. In addition, since 2005 we prospectively used single-antigen beads (SA beads) on the Luminex platform for immunological risk stratification (i.e., virtual crossmatch) with a cutoff of 500 MFI for positivity [17]. If current or historic donor-specific HLA antibodies (HLA-DSA) were present and the current CDC crossmatch was negative, the transplantation was performed, but considered as high risk, and an induction therapy with anti-thymocyte globulin (ATG) as well as intravenous immunoglobulins was given [18]. In the absence of HLA-DSA, basiliximab was used as induction therapy, and steroids were withdrawn beyond month 3 posttransplant, if no rejection had occurred [19]. In the third era (2015–2022; n = 580), donation after circulatory death (DCD) donors became more prevalent, and in general the acceptance criteria for allograft recipients were widened.

### Definition of Recipient Age Groups for the Analysis

The age of the recipients changed significantly from 1995 to 2022. Based on the age distribution in this cohort, we divided the patients arbitrarily into three age groups: (i) young patients having an age ≤40 years, (ii) middle-aged patients having an age of 41–60 years and (iii) elderly patients having an age >60 years.

### Data Collection

Clinical and histological data were prospectively collected into a research database. From 1995 to 2022 the clinical indication to perform an allograft biopsy did not change (i.e., declining allograft function, unsatisfactory allograft function, delayed graft function, proteinuria and/or glomerular hematuria). In addition, from 2001 until September 2017, surveillance biopsies were scheduled at 3 and 6 months posttransplant as a clinical routine. Since October 2017, surveillance biopsies at 3 and 6 months are only performed in patients with elevated urine CXCL10 [20]. The extent of HLA typing varied from 1995 to 2022, but the loci A/B/DR were available for all transplantations. As mentioned above, since 2005 the presence/absence of pretransplant HLA-DSA defined by SA beads was determined prospectively. Furthermore, for the era 1995–2004, we retrospectively assessed pretransplant HLA-DSA by SA beads in 349/570 (61%) transplantations in the context of a previous study [21].

### Assignment of Causes of Graft Loss

Graft losses were either classified as death with graft function or graft failure. Causes for graft failure were assessed by reviewing clinical data and histological findings from surveillance and/or indication biopsies or from transplant nephrectomies. Graft failures were assigned to one of eight categories: 1) rejection, 2) multifactorial with rejection [i.e., various hits including biopsy-proven rejection episodes], 3) multifactorial without rejection [i.e., various hits, but never biopsy-proven rejection], 4) recurrent or *de novo* glomerulonephritis, 5) surgical, 6) infection, 7) other causes, 8) unknown. The cause of death was assigned to one of five categories based on known medical diseases and the final event leading to death: 1) cardiovascular, 2) infection, 3) malignancy, 4) other causes, 5) unknown. To reduce the number of deaths classified as ‘unknown’, the treating physician’s presumed cause of death was used for the assignment. For example, if a patient died at home without any further clinical information, but the treating physician presumed a sudden cardiac death due to known heart disease, the cause of death was assigned as “cardiovascular.”

### Statistical Analysis

Categorical data are presented as counts and/or percentages and compared by Pearson’s chi-squared test. Continuous data are shown as median and interquartile ranges (IQR) and compared by Wilcoxon rank sum test. Survival curves were generated with the Kaplan Meier method and groups compared using the log-rank test. We used multivariable Cox proportional hazard models to investigate parameters associated with death or graft failure, respectively. All pretransplant available parameters were included in the models. A p-value <0.05 was considered to indicate statistical significance. Data were analyzed using JMP Pro Version 17 software (SAS institute Inc., Cary, NC, United States).

## Results

### Donor and Recipient Characteristics

The baseline characteristics of the 1743 transplantations are summarized in Table 1. There were 570 transplantations in the era 1995–2004, 593 transplantations in the era 2005–2014, and 580 transplantations in the era 2015–2022. As expected for a time span of 28 years, there were many significant differences among the recipient and donor characteristics in the three eras related to medical developments and change of policies. Deceased donors accounted for 52%–59% of all transplantations (p = 0.03), and the proportion of DCD donors increased from 2% to 26% (p < 0.0001). The median donor age increased from 50 to 57 years (p < 0.0001). The median number of HLA-A/B/DR mismatches and the frequency of retransplants was similar in the three eras (p = 0.13 and p = 0.10, respectively). The proportion of transplantations performed in the presence of HLA-DSA decreased from 21% to 12% (p = 0.0002).

**TABLE 1:** Baseline characteristics.

Parameter	1995–2004 (n = 570)	2005–2014 (n = 593)	2015–2022 (n = 580)	p-value^a^
Recipient sex female	228 (40%)	188 (32%)	204 (35%)	0.01
Recipient age	51 (40–59)	55 (44–63)	55 (44–63)	<0.0001
- Age >60 years	119 (21%)	196 (33%)	196 (34%)	<0.0001
- Age >70 years	9 (2%)	27 (5%)	40 (7%)	<0.0001
Donor age	50 (39–60)	54 (44–63)	57 (47–65)	<0.0001
- Age >60 years	131 (23%)	210 (35%)	219 (38%)	<0.0001
- Age >70 years	27 (5%)	61 (10%)	55 (9%)	0.0006
Deceased donor	298 (52%)	308 (52%)	342 (59%)	0.03
- DCD among deceased donor organs	6 (2%)	2 (0.6%)	90 (26%)	<0.0001
Renal disease				<0.0001
- Glomerulonephritis	200 (35%)	208 (35%)	195 (34%)	
- ADPKD	78 (14%)	115 (19%)	108 (19%)	
- Other nephropathies	63 (11%)	66 (11%)	85 (15%)	
- Unknown nephropathy	71 (12%)	67 (11%)	52 (9%)	
- Diabetic nephropathy	70 (12%)	62 (11%)	52 (9%)	
- Vascular/hypertensive nephropathy	22 (4%)	49 (9%)	55 (9%)	
- Interstitial nephritis	66 (12%)	26 (4%)	33 (5%)	
HLA mismatches [A/B/DR]	4 (3–5)	4 (3–5)	4 (3–5)	0.13
Retransplant	85 (15%)	103 (17%)	74 (13%)	0.10
HLA-DSA	73/349^b^ (21%)^c^	108 (18%)^d^	67 (12%)^d^	0.0002
ABOi living donor	-	58/285 (20%)	45/238 (19%)	<0.0001
Induction therapy	n = 473^b^			<0.0001
- basiliximab	137 (29%)	453 (76%)	453 (78%)	
- ATG	150 (32%)	125 (21%)	89 (15%)	
- None	186 (39%)	15 (3%)	38 (7%)	
Mainenance immunsuppression				<0.0001
- CyA based	365 (64%)	3 (1%)	2 (0.5%)	
- Tac-MMF	15 (3%)	466 (79%)	576 (99%)	
- Tac-Aza	120 (21%)	-	2 (0.5%)	
- Tac-mTOR	-	107 (18%)	-	
- mTOR based	65 (11%)	9 (1%)	-	
- Other	5 (1%)	8 (1%)	-	

^a^ The p-value was calculated using Pearson’s chi-squared or Wilcoxon rank sum tests across the three eras (no comparison between individual eras was performed).

^b^ If the parameter was not available for all patients, the number of retrieved data are given.

^c^ Retrospective analysis.

^d^ Prospectively assessed.

DCD, donation after circulatory death; ADPKD, autosomal dominant polycystic kidney disease; HLA-DSA, donor-specific HLA, antibodies.

### Evolution and Grouping of Recipient Age

**FIGURE 2:**
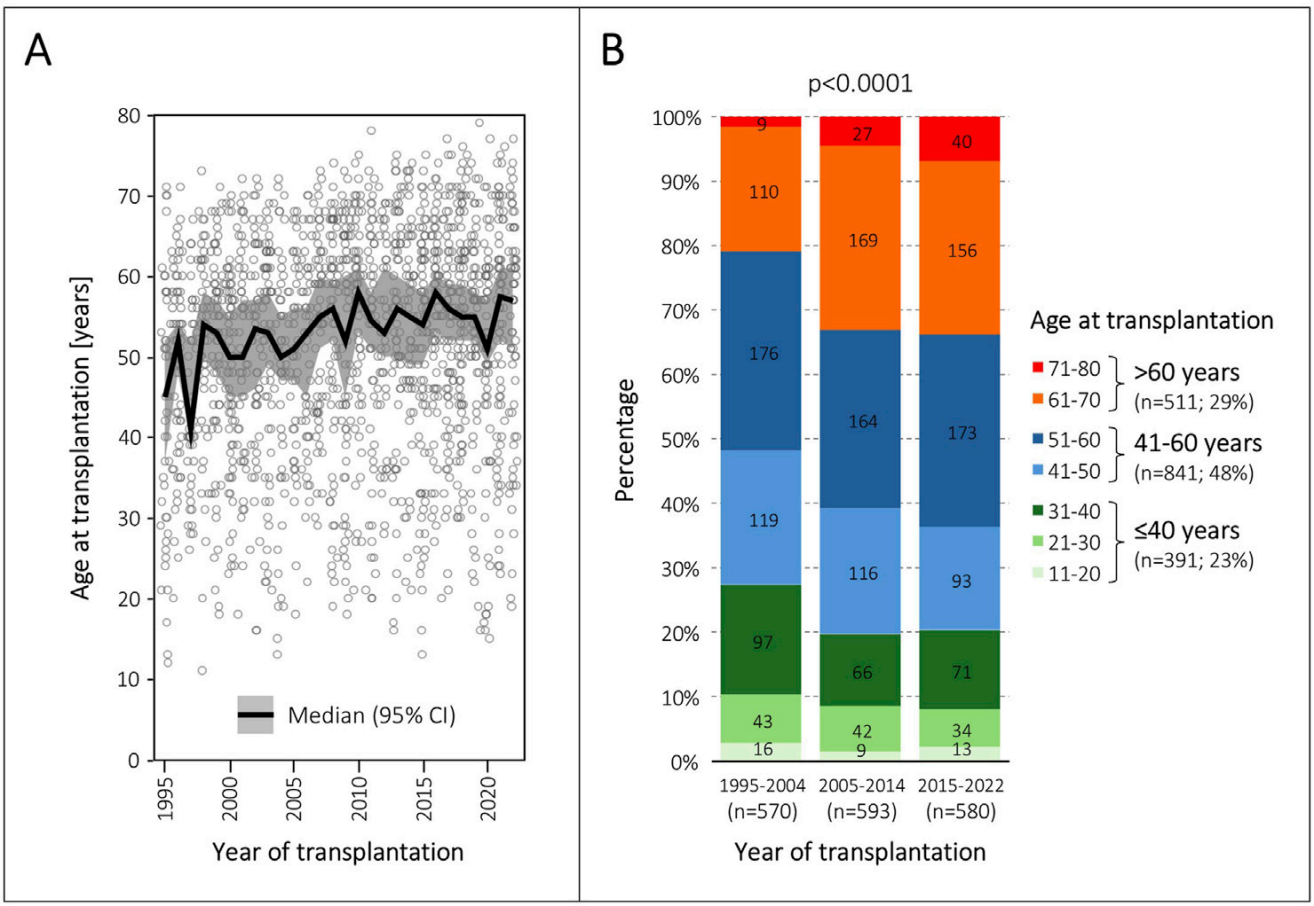
Evolution of patient age at transplantation. **(A)**, Annual evolution of median age and its 95% confidence interval (CI) at transplantation from 1995 to 2022. The grey circles represent individual patients. **(B)**, Distribution of age groups in the three transplantation eras.

The median recipient age in the three eras increased from 51 to 55 years (p < 0.0001). The annual evolution of the median recipient age and its 95% confidence interval is shown in Figure 2A. Most importantly, the proportion of recipients older than 60 years increased from 21% to 34% (p < 0.0001), and the proportion of recipients older than 70 years from 2% to 7% (p < 0.0001) (Table 1). The distribution of age groups by decades in the three eras is detailed in Figure 2B. According to our arbitrarily defined age thresholds, 391 (23%) patients were ≤40 years old, 841 (48%) patients were 41–60 years old, and 511 (29%) patients were >60 years old (Figure 2B).

### Overall Causes of Graft Loss

**FIGURE 3:**
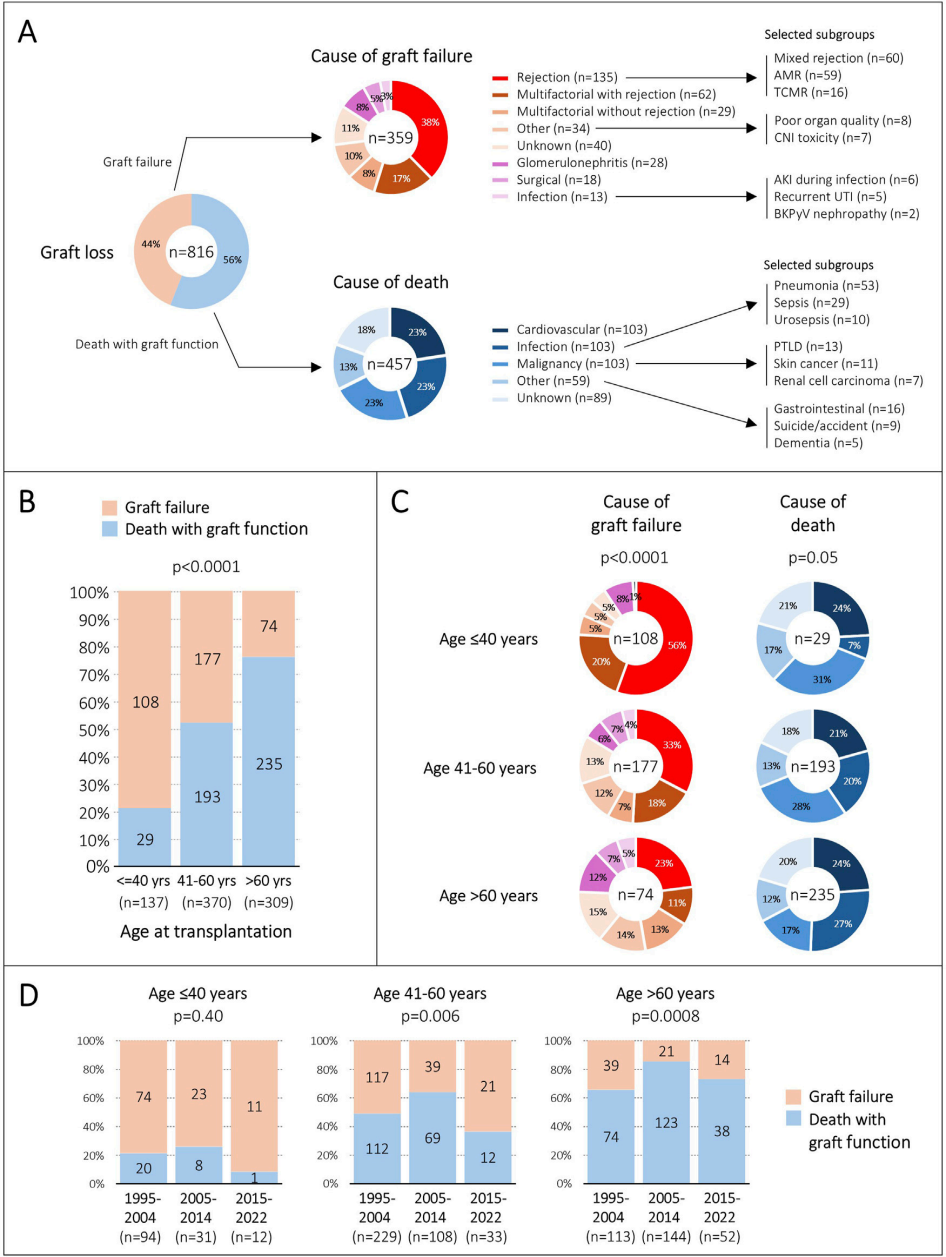
Causes of graft loss. **(A)**, Overall contribution of death and graft failure on graft loss, as well as their specific causes. **(B)**, Contribution of death and graft failure on graft loss, stratified by age group at transplantation. **(C)**, Frequency of causes of death and graft failure among the three age groups. **(D)**, Distribution of death and graft failure on graft loss during the three eras, stratified by age group at transplantation. AMR, antibody-mediated rejection; TCMR, T cell-mediated rejection; CNI, calcineurin-inhibitor; AKI, acute kidney injury; UTI, urinary tract infection; BKPyV, BK polyomavirus; PTLD, posttransplant lymphoproliferative disease.

Overall, 816/1743 (47%) allografts were lost. Deaths with graft function accounted for 56% of graft losses, whereas graft failures accounted for 44% (Figure 3A).

Cardiovascular events, infections and malignancies were equally frequent as the cause of death, each contributing 23%. Thirteen percent of deaths could not be assigned to one of these categories and were summarized as ‘other causes’. This included many different individual entities such as gastrointestinal diseases, dementia, suicide, and accidents. The cause of death remained unknown in 18% of cases. Almost all these patients died at home without a prior acute illness, suggesting that many deaths could be related to acute cardiovascular events. Within all major categories, several subgroups with more granular entities were seen. Some of these subgroups of particular interest are summarized in Figure 3A.

Rejection was the most frequent cause of graft failure (38%), followed by multifactorial hits including rejection (17%). Therefore, rejection contributed exclusively or partially to graft failure in 55% in our cohort. The cause of graft failure could not be assigned to any category in 11%. These were mostly cases having slowly deteriorating allograft function without any clearly attributable diseases and without histological investigations. Within the rejection category, mixed rejection and isolated antibody-mediated rejection (AMR) were equally frequent, each accounting for 44%. In all major categories, several subgroups with more granular entities were observed. Some of these subgroups of particular interest are summarized in Figure 3A.

### Causes of Graft Loss in the Three Age Groups

The proportion of death with graft function and graft failure contributing to graft loss was significantly different among the three age groups. While graft failure accounted for 79% of all graft losses in patients ≤40 years old, death with graft function was responsible for graft loss in 76% of patients with an age >60 years (p < 0.0001) (Figure 3B).

Next, we compared the causes of graft failures among the three age groups, and we noticed significant differences. Rejection alone or in combination with other hits accounted for 76% of graft failures in young patients, for 51% in middle-aged patients, and for only 34% in elderly patients (p < 0.0001) (Figure 3C).

Interestingly, the causes of death were not significantly different among the three age groups (p = 0.05) (Figure 3C). However, we observed a trend towards more infection-related deaths with increasing age (7% vs. 20% vs. 27%).

Over the three eras, we observed some differences regarding the contribution of death with graft function and graft failure on graft loss among the age groups. However, graft failure accounted for 74%–92% of graft losses in young patients, whereas death with graft function was responsible for 65%–85% of graft losses in elderly patients (Figure 3D).

### Evolution of Patient and Graft Survival in the Three Age Groups

**FIGURE 4:**
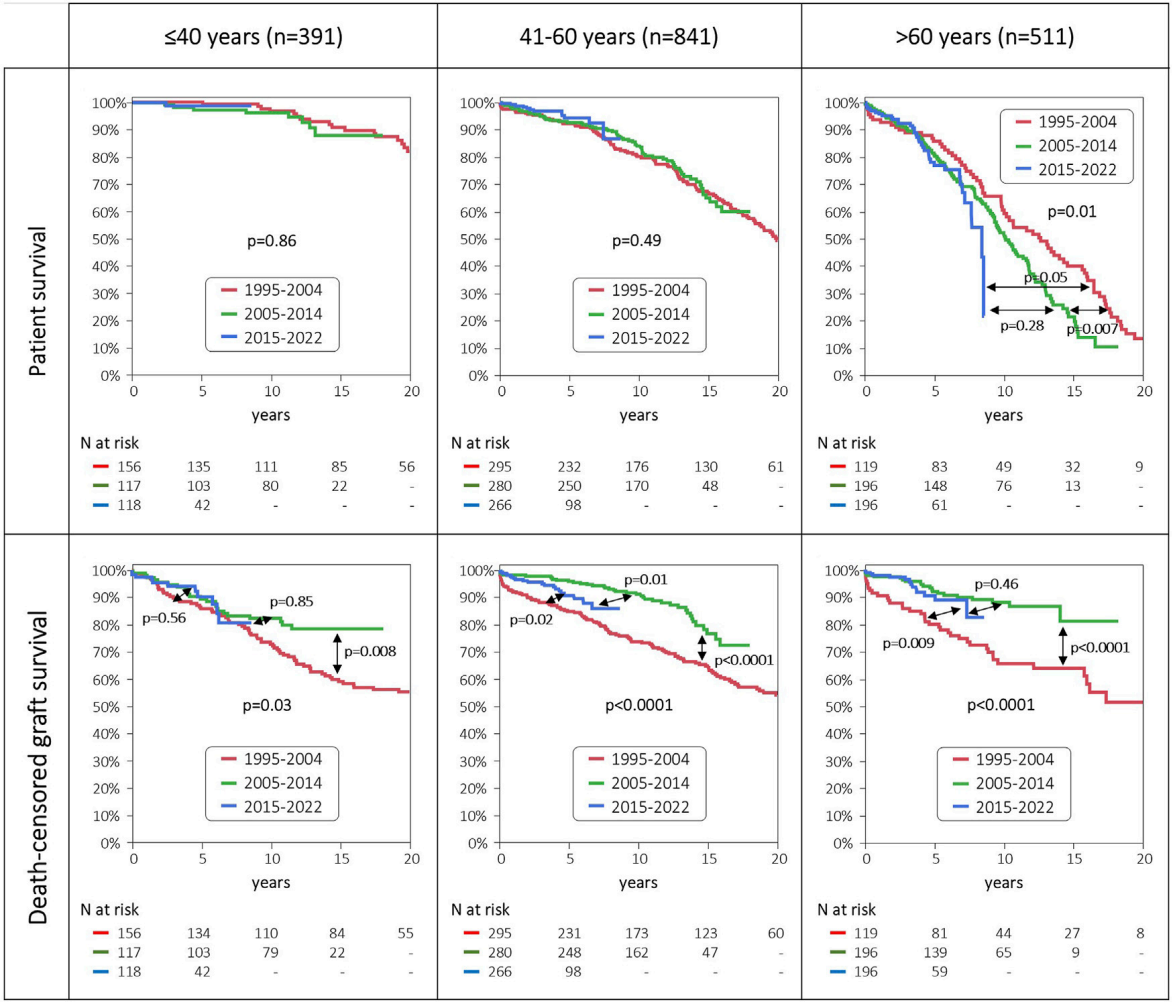
Patient and death-censored graft survival, stratified by age groups and era of transplantation.

Patient survival remained high and unchanged throughout the three eras among the young and middle-aged patient groups. By contrast, in the group of elderly patients we observed a slight increase in the 1-/3-year patient survival in the two recent eras, but beyond the fifth year posttransplant patient survival diminished from the 1995–2004 era to the 2005–2014 era, and the 2015–2022 era (Figure 4).

Death-censored graft survival improved dramatically in the middle-aged and elderly patient groups from the 1995–2004 era to the 2005–2014 era. In the most recent era from 2015 to 2022 no further improvement was observed, and even a slight decrease was noticed in the middle-aged patient group. In sharp contrast, we saw no improvement in 5-/10-year death-censored graft survival in the young patient group throughout the three eras.

### Multivariable Analyses

In the multivariable Cox model, patient age was a strong and independent risk factor for death (hazard ratio 2.32 per decade [95% CI: 2.04–2.64]; p < 0.0001). Other statistically significant risk factors were diabetic nephropathy as primary kidney disease, deceased donor as the organ source, and the presence of HLA-DSA (Table 2).

**TABLE 2:** Multivariable Cox proportional hazard model of patient survival.

Parameter	HR (95% CI)	p-value	Logworth [−log_10_ (p-value)]
Recipient age per decade	2.32 (2.04–2.64)	<0.0001	37.121
Renal disease		<0.0001	4.682
- Glomerulonephritis	Reference		
- ADPKD	1.16 (0.84–1.60)	0.351	
- Other nephropathies	1.40 (0.92–2.12)	0.11	
- Unknown nephropathy	0.96 (0.66–1.39)	0.83	
- Diabetic nephropathy	2.28 (1.64–3.15)	<0.0001	
- Vascular/hypertensive nephropathy	1.49 (1.00–2.18)	0.05	
- Interstitial nephritis	1.14 (0.72–1.82)	0.57	
Donor source		0.0001	3.878
- Living donor	Reference		
- Deceased donor, DBD	1.65 (1.30–2.09)	<0.0001	
- Deceased donor, DCD	1.90 (0.94–3.86)	0.07	
HLA-DSA	1.41 (1.05–1.91)	0.02	1.627
Maintenance immunosuppression		0.05	1.340
- Tac-MMF	Reference		
- CyA based	0.62 (0.44–0.88)	0.01	
- mTOR based	0.58 (0.34–0.98)	0.04	
- Tac-mTOR	0.97 (0.68–1.39)	0.88	
- Tac-Aza	1.02 (0.71–1.48)	0.91	
Recipient sex female	0.86 (0.68–1.08)	0.20	0.703
Donor age per decade	1.03 (0.96–1.09)	0.43	0.365
ABOi living donor	0.84 (0.46–1.52)	0.56	0.248
HLA mismatches [A/B/DR]	1.18 (0.78–1.81)	0.58	0.234
Repeat transplant	1.02 (0.95–1.10)	0.83	0.081

DBD, donation after brain death; DCD, donation after circulatory death; ADPKD, autosomal dominant polycystic kidney disease; HLA-DSA, donor-specific HLA, antibodies.

Statistically significant and independent risk factors for death-censored graft survival were donor age, Cyclosporin- or mTOR-based maintenance immunosuppression, deceased donor as organ source, presence of HLA-DSA, and HLA mismatches. In addition, patient age was inversely associated with death-censored graft survival (hazard ratio 0.84 per decade [[95% CI: 0.75–0.93]; p = 0.0007) (Table 3).

**TABLE 3:** Multivariable Cox proportional hazard model of death-censored graft survival.

Parameter	HR (95% CI)	p-value	Logworth [-log_10_(p-value)]
Donor age per decade	1.20 (1.10–1.30)	<0.0001	4.803
Maintenance immunosuppression		0.0001	4.175
- Tac-MMF	Reference		
- CyA based	1.95 (1.41–2.70)	<0.0001	
- mTOR based	2.26 (1.47–3.47)	0.0002	
- Tac-mTOR	0.79 (0.44–1.39)	0.41	
- Tac-Aza	1.51 (0.98–2.31)	0.06	
Donor source		0.0001	4.156
- Living donor	Reference		
- Deceased donor, DBD	1.86 (1.40–2.45)	<0.0001	
- Deceased donor, DCD	0.76 (0.42–3.28)	0.76	
HLA-DSA	1.82 (1.33–2.49)	0.0002	3.796
Recipient age per decade	0.84 (0.75–0.93)	0.0007	3.186
Renal disease		0.01	1.955
- Glomerulonephritis	Reference		
- ADPKD	0.80 (0.53–1.21)	0.29	
- Other nephropathies	1.26 (0.88–1.80)	0.22	
- Unknown nephropathy	0.50 (0.30–0.84)	0.01	
- Diabetic nephropathy	1.17 (0.75–1.82)	0.48	
- Vascular/hypertensive nephropathy	1.33 (0.83–2.13)	0.24	
- Interstitial nephritis	0.65 (0.39–1.10)	0.11	
HLA mismatches [A/B/DR]	1.10 (1.01–1.21)	0.03	1.520
Repeat transplant	1.22 (0.88–1.70)	0.24	0.616
Recipient sex female	0.89 (0.68–1.16)	0.37	0.430
ABOi living donor	0.76 (0.34–1.68)	0.50	0.304

DBD, donation after brain death; DCD, donation after circulatory death; ADPKD, autosomal dominant polycystic kidney disease; HLA-DSA, donor-specific HLA, antibodies.

### Subgroup Analyses of Recipients Older Than 70 years

Seventy-six patients were older than 70 years at the time of transplantation (range 71–79). The one-, five-, and 10-year patient survival were 89%, 68%, and 40%. The one-, five-, and 10-year death-censored graft survival were 97%, 89%, and 82%. We observed no differences among the three eras regarding patient survival (p = 0.99) and death-censored allograft survival (p = 0.81) (data not shown). Eight grafts were lost, only one due to rejection (13%). Thirty-four patients died. The causes of death were not different compared to patients with 60–70 years of age (p = 0.15) (data not shown).

## Discussion

The key observation of this study was that the causes of graft loss are strongly age-dependent. While 79% of young patients (≤40 years) lose their transplant due to rejection-induced graft failure, 76% of older patients (>60 years) experience graft loss due to death with graft function. This proportion remained stable during the study period from 1995 to 2022.

In line with other studies, our recipient population has markedly changed over the last three decades [3, 4]. The proportion of patients being older than 60 years at the time of transplantation has significantly increased from 21% in the first era to 33%–34% in the later two eras.

Consistent with findings from other studies, the main causes of death were malignancies, infection diseases and cardiovascular events, all accounting for about a quarter of cases [4, 10, 11]. The lower percentage of cardiovascular death in Van Loon et al. may be explained by their higher proportion of deaths assigned as ‘unknown’ [12]. Indeed, Mayrdorfer et al. and we classified sudden death in a patient with known cardiovascular diseases as “cardiovascular” rather than “unknown” [10]. The distribution of the causes of death did not significantly change when comparing across different age groups or eras. However, there was a trend towards increasing infection-related deaths in elderly recipients, suggesting a state of overimmunosuppression in this patient group.

The causes of graft failure were highly dependent on recipient age. While other cohort studies have focused more on the causes of early versus late graft failure, our approach was to investigate distinct recipient populations [12, 14]. Overall, the total proportion of rejection-related graft failures in our study was 55% (i.e., 38% rejection as the main cause, 17% multifactorial with rejection). This is consistent with Mayrdorfer et al reporting an overall proportion of 65% (i.e., 35% rejection as the primary cause, 30% rejection as the secondary cause). Interestingly, primarily calcineurin-inhibitor (CNI) toxicity-related graft failure was rare (7/359; 2%), which is in line with other studies (0.6%–0.7%) [13, 14]. However, the impact of CNI-toxicity might be underestimated and could contribute to graft failures classified as “non-specific chronic injury” as in the study by Van Loon et al. (21%; [12]), or was assigned as a secondary cause of graft failure as in the study by Mayrdorfer et al (21%; [14]), or is part of the category “multifactorial without rejection” as in our study (8%).

Patient survival is very high in young and middle-aged patients, and it did not improve from 1995 to 2022. By contrast, we observed a decrease in patient survival in elderly patients, likely related to a more liberal policy to accept more elderly patients with significant comorbidities for renal transplantation. In addition, it suggests that we might have reached the limit of current treatment concepts in this patient population, and it calls for alternative strategies, such as lower and/or less toxic immunosuppression.

The introduction of Tacrolimus/MMF-based immunosuppression and a better immunological risk stratification both led to the remarkable improvement in death-censored graft survival from the 1995–2005 era to the 2005–2015 era. However, young patients seem to benefit the least from these advances with an almost unchanged and high proportion of rejection-related graft failures [3, 9]. Both a stronger immune response in general and non-adherence are considered as the main contributing factors [11, 22]. In the other two age groups there is no further improvement in death-censored graft survival during the last era from 2015 to 2022. This might be attributed to an expansion of donor acceptance criteria.

The strong impact of recipient age on the causes of graft loss has important implications. First, it highlights that pertinent endpoints in studies should ideally be age-dependent. For young patients, the occurrence of rejection and graft failure due to rejection are most important, while for elderly patients quality of life, immunosuppression-related toxicity, and patient survival are more reasonable endpoints. Second, patient management could be adapted according to the age group and its most important associated risks. For example, young patients might benefit from a denser immunological surveillance, the implementation of stronger immunosuppressive regimens, strengthening of adherence and allocation of well HLA-matched organs partially compensating for non-adherence [23]. Elderly patients might be managed by a rather lower immunosuppression and an emphasis on cardiovascular risk factors and the prevention/surveillance of infections and cancer.

The Eurotransplant senior program (ESP) was established in 1999 and had the aim to allocate kidneys from donors ≥65 years to recipients ≥65 years. These transplants were mostly performed locally to reduce cold ischemia time and HLA matching was disregarded [24]. Although the immune system ages like all other organs, rejection is still a concern in elderly patients. Interestingly, the ESP found a superior patient and allograft survival, if full HLA-DR matching was enforced [25]. This suggests that HLA-matching has also benefits in elderly patients, potentially allowing for a lower overall level of immunosuppression without increasing the risk of rejection. However, a personalized approach to immunosuppression incorporating the immunological background, rejection episodes, biomarkers and the presence of side effect of immunosuppression is still preferable [26, 27].

Despite a higher risk of death in elderly patients, many seem to benefit from renal transplantation, especially regarding the quality of life compared to dialysis. Unfortunately, an adequate comparison of mortality and morbidity between patients receiving a transplantation and patients on dialysis is very challenging due to various biases (e.g., selection bias) [12, 28, 29].

The advantages of our study are the larger size of investigated graft losses compared to other cohorts and the focus on the impact of recipient age on graft losses [10–13]. Furthermore, the inclusion of transplantations performed between 1995 and 2022 allowed to assess changes over almost 30 years. Finally, the results are supported by univariate and multivariate analyses.

We acknowledge several important limitations of this study. First, the results of our single center study may lack generalizability, especially because the population consisted almost exclusively of Caucasian ethnicity. Second, the retrospective study design does not allow to define causal relationships, although data were collected prospectively. Third, the long period of observation is introducing potential biases related to changes in management policies which we cannot account for.

In conclusion, the causes of graft loss are strongly age-related. This might have significant implications for the design and endpoint definition in clinical studies, as well as for individual patient management.

## Data Availability

The data analyzed in this study is subject to the following licenses/restrictions: The data that support the findings of this study are available from the corresponding author upon reasonable request. Requests to access these datasets should be directed to Stefan Schaub, stefan.schaub@usb.ch.
